# Retrospective Clinical and Radiographic Outcomes of a Cageless Tibial Tuberosity Advancement Technique in Small-Breed Dogs

**DOI:** 10.3390/ani16081212

**Published:** 2026-04-16

**Authors:** William McCartney, Christos Yiapanis, Ciprian Ober, Amarildo Gjeli, Denis Gaceu, Joshua Milgram

**Affiliations:** 1 North Dublin Orthopaedic Animal Hospital, Warrenhouse Rd 38, Baldoyle, D13 K5H0 Dublin, Ireland; billymccartney@gmail.com; 2 Department of Veterinary Medicine, School of Veterinary Medicine, University of Nicosia, 2414 Nicosia, Cyprus; drcy@cyvets.com; 3 Department of Surgery and Intensive Care, Faculty of Veterinary Medicine, University of Agricultural Sciences and Veterinary Medicine, Calea Manastur 3-5, 400372 Cluj-Napoca, Romania; gaceudenis@gmail.com; 4 CYVETS, Leof. T. Papadopoulou 138, 8025 Paphos, Cyprus; amarildogj@cyvets.com; 5 Department of Small Animal Surgery, Koret School of Veterinary Medicine, The Robert H. Smith Faculty of Agriculture, Food & Environment, The Hebrew University of Jerusalem, P.O. Box 12, Rehovot 76100, Israel; josh.milgram@mail.huji.ac.il

**Keywords:** bone graft, cranial cruciate ligament rupture, dog, pin, screw, small-breed dogs, tibial tuberosity advancement

## Abstract

Cranial cruciate ligament rupture is a common orthopedic condition in dogs, and tibial tuberosity advancement is a well-established surgical treatment. The aim of this retrospective study was to evaluate the short-term clinical and radiographic outcomes of a cageless tibial tuberosity advancement technique in small-breed dogs. Medical records of 63 dogs (77 stifles) treated using this technique were reviewed. Fixation was achieved using three construct types: a screw–pin construct (the majority of cases), a screw-only construct, or a screw combined with two pins. Due to small subgroup sizes, fixation-type outcomes were primarily analyzed descriptively. Clinical and radiographic evaluations were performed immediately after surgery and at eight weeks postoperatively. Clinical outcomes were graded based on limb function, and radiographic bone healing was scored using a standardized scale. Postoperative complications were recorded and analyzed in relation to patient and procedural variables. No intraoperative complications were observed, while postoperative complications occurred in 27% of dogs and were predominantly minor and implant-related. Lameness scores improved significantly over the follow-up period. All treated stifles demonstrated stable implants, maintained advancement, and satisfactory bone healing. The use of bone graft material appeared to be associated with fewer complications and more favorable clinical outcomes; however, this observation should be interpreted with caution given the retrospective and non-randomized design of the study. In this retrospective case series, cageless tibial tuberosity advancement using screw-based fixation (predominantly screw–pin constructs) was associated with favorable short-term clinical and radiographic outcomes. These findings should be considered preliminary and limited to short-term evaluation, given the retrospective design, absence of a control group, and relatively short follow-up period. Further prospective studies with larger populations, standardized outcome measures, and longer follow-up are warranted to confirm these findings.

## Simple Summary

Rupture of the cranial cruciate ligament is a common cause of hindlimb lameness in dogs and is often managed with surgery. Tibial tuberosity advancement is a widely used surgical technique that stabilizes the knee joint by changing its biomechanics. Several variations in this technique exist, differing mainly in the type and number of implants used. This study evaluated a simplified tibial tuberosity advancement technique that does not use a cage and relies on a single screw, with or without an additional pin, in small-breed dogs. Medical records and follow-up examinations of 63 dogs were reviewed to assess clinical recovery, radiographic bone healing, and complications over an eight-week period after surgery. Most dogs showed marked improvement in limb use, and bone healing was satisfactory in all treated joints. Complications were mainly minor and related to implant irritation. The use of bone graft material was associated with fewer complications and better clinical outcomes compared with cases in which bone graft was not used. These results suggest that this simplified technique can be a safe and effective surgical option for treating cranial cruciate ligament rupture in small-breed dogs.

## 1. Introduction

Tibial tuberosity advancement (TTA) is a commonly used surgical procedure for the treatment of cranial cruciate ligament rupture in dogs [1]. Conservative management of cranial cruciate ligament rupture in small-breed dogs has been recommended in some reports [2,3]; however, surgical procedures similar to those performed in large-breed dogs are frequently advocated, as they are associated with a faster return to limb function [4,5,6,7,8,9]. The original TTA technique described by Montavon et al. [10] involved a complete osteotomy of the tibial tuberosity, which was stabilized with a plate. Subsequent modifications of this technique were aimed at reducing the number of implants, procedural cost, surgical time, and the risk of postoperative complications such as deep infection.

Modified TTA techniques typically eliminate the need for a plate by using an incomplete osteotomy of the tibial tuberosity, leaving a cranial cortical bone bridge to maintain stability. In all TTA techniques, a spacer is placed between the tibial plateau and the tibial tuberosity to maintain tibial tuberosity advancement [11].

Several modified techniques have been described, including the modified Maquet technique [12], TTA Rapid [13,14], and other variations [11,15,16,17,18,19], with recent reviews highlighting the diversity of implant configurations and clinical outcomes [20]. In addition to technical modifications aimed at reducing implant number and surgical complexity [11,12,13,14,15,16,17,18,19], increasing attention has been directed toward objective assessment of functional outcomes following cranial cruciate ligament surgery in dogs. Force plate and pressure-sensitive walkway analyses have been widely used to quantify limb loading and provide objective evaluation of postoperative function [21], although such methods are not always available in clinical practice. Furthermore, morphometric and biomechanical factors influencing surgical planning and outcomes have been investigated, highlighting the complexity of load distribution and stabilization mechanisms in the canine stifle [22]. Within this context, simplified fixation strategies should be evaluated not only in terms of clinical feasibility but also with consideration of their biomechanical rationale and functional outcomes.

In the TTA Rapid technique, implants used for dogs weighing ≤ 4 kg are scaled-down versions of those designed for medium- and large-breed dogs [22]. TTA Rapid has been successfully applied in small-breed dogs with cranial cruciate ligament rupture, demonstrating complication rates comparable to those reported in larger breeds and high levels of owner satisfaction [9]. However, the mechanical forces acting on the tibial tuberosity in small-breed dogs remain poorly characterized, and the necessity for implant configurations designed for larger dogs may be questioned. In this context, fixation using a single screw, with or without an additional distal pin, may be sufficient to maintain the tibial tuberosity in its advanced position in small-breed dogs.

The aim of this retrospective case series was to describe a simplified cageless tibial tuberosity advancement technique using screw or screw–pin fixation and to evaluate its short-term clinical and radiographic outcomes, as well as perioperative complications, in a population of small-breed dogs.

## 2. Materials and Methods

### 2.1. Animals

A total of 63 small-breed dogs (defined in this study as dogs weighing ≤ 15 kg) representing 77 stifles underwent cageless tibial tuberosity advancement surgery. Cases were collected retrospectively from two veterinary referral centers participating in the study. Consecutive cases with complete medical records and follow-up data were included to minimize selection bias. Of these, 49 dogs underwent unilateral surgery and 14 dogs underwent bilateral surgery; among bilateral cases, 8 procedures were performed simultaneously and 6 were staged. Stabilization was most commonly achieved using a screw–pin construct. The described fixation concept was biomechanically evaluated in vitro prior to its clinical application, as previously reported [14]. In a limited number of cases, alternative constructs were used, including screw-only fixation or screw combined with two pins.

Dogs presenting with cranial cruciate ligament disease in the contralateral limb underwent either simultaneous or staged bilateral procedures. Data collected for each case included breed, age, sex, neutering status, body weight, implant configuration used to stabilize the tibial tuberosity, and clinical outcomes. Formal approval from an institutional ethics committee was not required because the study consisted of a retrospective analysis of clinical cases treated as part of routine veterinary practice and did not involve experimental procedures. Written informed consent was obtained from all owners for participation in the study and for publication of relevant clinical data and images.

Preoperative mediolateral radiographs of the stifle joint in extension were obtained for surgical planning and calculation of the required tibial tuberosity advancement [10]. The common tangent method was used to determine the degree of advancement in each case. Using a commercially available surgical planning template, the thickness of the cranial tibial cortex, the thickness of the cranial tibial cortex at the distal end of the planned osteotomy was measured to ensure preservation of adequate cortical bone distal to the osteotomy and to minimize the risk of iatrogenic cortical damage.

### 2.2. Preoperative Considerations

Anesthetic management was individualized for each patient. Dogs were premedicated with low doses of medetomidine (5 µg/kg IV) in combination with butorphanol (0.2 mg/kg IV) or methadone hydrochloride (0.5 mg/kg IV), and ketamine (1 mg/kg IV). Anesthesia was induced with propofol (2 mg/kg IV) and maintained with isoflurane in oxygen. Cefazolin (22 mg/kg IV) was administered approximately 40 min prior to skin incision. Lactated Ringer’s solution was administered intravenously throughout anesthesia at a rate of 5 mL/kg/h.

### 2.3. Surgical Technique

All surgical procedures were performed by experienced veterinary surgeons familiar with tibial tuberosity advancement techniques and following a standardized surgical approach. General procedure and osteotomy technique were standardized, while the implants used (including the use of an additional pin or bone graft) were determined intraoperatively at the surgeon’s discretion.

Mini-arthrotomy or arthroscopy was performed to inspect the menisci and cruciate ligaments in joints diagnosed with complete or presumptively complete cranial cruciate ligament rupture, confirmed by clinical instability (positive cranial drawer or tibial thrust).

Debridement of ligament remnants and partial meniscectomy were performed when required. Meniscal release was not performed in any case, and the Maquet hole was omitted from the procedure. Before osteotomy, a small pilot hole was predrilled using a 1.1 or 1.2 mm Kirschner wire, depending on patient size, to a depth of approximately 1 mm, immediately distal to the insertion of the patellar ligament. The tibial tuberosity osteotomy was performed using a custom-designed TTA guide (Rita Leibinger, GmbH & Co. KG, Mühlheim an der Donau, Germany) and a veterinary oscillating saw fitted with a fine-toothed stainless-steel blade (Synthes Vet, Oberdorf, Switzerland). A dedicated manual spreader was used to advance the tibial tuberosity to the preoperatively calculated distance. The screw tunnel was drilled using a 1.8 mm drill bit. Drilling was initiated at the predrilled hole, and the drill was oriented perpendicular to the long axis of the tibia and advanced until it exited the caudal cortex distal to the tibial plateau.

A 2.4 mm titanium self-tapping cruciform-head screw (DePuy Synthes Vet, West Chester, PA, USA) was inserted to engage both the cranial and caudal cortices. An additional distal pin was placed in 56 dogs. The decision to add a pin was made intraoperatively at the surgeon’s discretion. The diameter of the screw (2.4 mm) was consistent across all cases, while screw length and pin diameter were selected according to patient size and the degree of advancement required. The dimensions of the fixation components, including screw length and pin diameter, were selected according to patient size and the degree of tibial tuberosity advancement required.

Although the standard fixation method consisted of a screw–pin construct, minor variations were applied in some cases according to intraoperative considerations. Specifically, screw-only fixation was used in a small number of stifles, while in two cases an additional stabilizing pin was placed to increase construct stability, resulting in a screw and two-pin construct.

Following implant placement, the blood clot within the osteotomy site was left undisturbed. In cases where a blood clot did not form spontaneously, small incisions were made in adjacent fat tissue to promote hemorrhage and clot formation within the osteotomy gap. Calcium phosphate bioceramic bone graft substitute was placed within the osteotomy gap in selected cases, at the surgeon’s discretion, to provide an osteoconductive scaffold to support bone formation within the osteotomy site. The decision to use a bone graft substitute or to place an additional distal pin were not standardized. Intraoperative assessment, including perceived construct stability, osteotomy gap characteristics, and patient size were considered, and bone graft substitute and additional implants was placed at the discretion of the operating surgeon.

Postoperative radiographs were obtained in all dogs to assess implant position, advancement maintenance, and the presence of fissures or fractures associated with the osteotomy.

### 2.4. Postoperative Management

Postoperative analgesia included firocoxib (5 mg/kg orally) or carprofen (4.4 mg/kg orally) once daily for 10 days. Gabapentin (10 mg/kg orally twice daily) and tramadol (3 mg/kg orally three times daily) were administered for the first five postoperative days and adjusted thereafter based on clinical assessment. This dosing regimen was used as part of a multimodal analgesic protocol and reflects the standard clinical practice of the participating centers. All owners were instructed to restrict activity for eight weeks postoperatively, and physiotherapy was recommended beginning two weeks after surgery to encourage limb function [23].

### 2.5. Clinical and Radiographic Evaluation

Clinical outcomes were classified into four categories based on postoperative limb function and the presence of complications: poor (non-use of the limb or major complications such as fixation failure), satisfactory (persistent intermittent lameness), good (infrequent intermittent lameness), and excellent (absence of observable lameness). In this context, the term “satisfactory” was used to indicate functional limb use with partial clinical improvement, although intermittent lameness persisted.

Outcome classification was based primarily on orthopedic examinations performed by a veterinary surgeon. In cases where a full in-person clinical evaluation was not available, owner-reported information obtained by telephone was used to complement the clinical assessment. All evaluations were performed using a consistent assessment framework across participating centers. Complications were classified as major when additional surgical intervention was required, and as minor when they resolved with conservative management [23]. All dogs underwent clinical and radiographic re-evaluation approximately eight weeks after surgery; owner-reported information obtained by telephone was used to complement the clinical assessment when necessary. Orthopedic evaluation included gait assessment, lameness scoring, inspection of the surgical site, and joint palpation. At the time of follow-up clinical examination none of the cases were receiving pain medication ensuring that lameness assessment was not influenced by ongoing analgesic treatment. Lameness was graded using a numerical scale from 0 (sound) to 4 (non-weight-bearing). Lameness scoring was performed both preoperatively and at the eight-week postoperative re-evaluation using the same scoring system. Clinical and gait assessments were performed by experienced veterinary surgeons involved in the study, following the same evaluation protocol across all cases.

Mediolateral and craniocaudal radiographs of the operated stifle were obtained at eight weeks postoperatively to assess implant position, maintenance of tibial tuberosity advancement, and osteotomy healing. The 8-week follow-up time point was selected as it represents a commonly used interval for assessing early osteotomy healing following tibial tuberosity advancement procedures, at which partial to complete bone bridging is typically expected [23]. The mediolateral projection allowed evaluation of implant placement, advancement maintenance, and bone bridging across the osteotomy site, while the craniocaudal projection permitted assessment of implant alignment and potential mediolateral displacement of the tibial tuberosity. In routine clinical practice at our institution, the craniocaudal projection is used interchangeably with the caudocranial view for postoperative assessment of the stifle. Radiographic bone healing was graded according to previously published criteria [23]: grade 0, no healing; grade 1, early bone production without bridging; grade 2, bone bridging proximal or distal to the screw; and grade 3, bone bridging proximal and distal to the screw. Healing grade 2 was defined as partial bone bridging visible either proximal or distal to the fixation screw, even when the osteotomy margins remained partially visible on mediolateral projection. Based on previous reports of tibial tuberosity advancement procedures, partial to complete bone bridging (healing grades 2–3) is typically expected within approximately eight weeks after surgery. All radiographs were evaluated by an experienced veterinary surgeon familiar with postoperative orthopedic imaging, and all assessments were performed by the same individual to ensure consistency.

### 2.6. Statistical Analysis

Associations between laterality, body weight, age, bone graft use, and the presence of meniscal injury with postoperative complications and clinical outcome were evaluated. Two primary outcomes were analyzed: occurrence of complications and clinical outcome category, both treated as categorical variables. Mean values and standard deviations were calculated for continuous variables. Associations between categorical variables were assessed using Fisher’s exact test. Continuous variables were assessed for approximate normality and compared between groups using an unpaired *t*-test. Statistical significance was set at *p* < 0.05.

Because fixation constructs differed in a limited number of cases, subgroup outcomes according to construct type (screw–pin, screw-only, screw with two pins) were evaluated descriptively. In addition, a sensitivity analysis was performed excluding cases treated with non-standard constructs (screw-only and screw with two pins) in order to verify whether the overall clinical outcomes differed when only the predominant screw–pin fixation was considered.

## 3. Results

A total of 77 stifles from 63 small-breed dogs were included in this retrospective case series. Detailed individual case information is provided in Supplementary Table S1. Dogs weighed ≤ 15 kg and represented a variety of small breeds. A screw–pin construct was used in 65/77 stifles (56 dogs), in which a distal pin was placed in addition to the screw. Bone graft material was used in 14 of 77 stifles (18%). Of the 14 dogs that underwent bilateral surgery, 8 procedures were performed simultaneously and 6 were staged. Meniscal pathology requiring debridement was identified intraoperatively in 7 of 77 stifles (9.1%). No intraoperative complications were recorded.

Postoperative complications occurred in 17 of 63 dogs (27%) and were classified as major (*n* = 5) or minor (*n* = 12). Minor complications consisted primarily of implant-related skin irritation associated with the pin (*n* = 5) and/or screw (*n* = 8) which resolved with conservative management. Implant removal was required in five cases due to persistent implant-related skin irritation or wound complications. Dehiscence of the surgical site associated with local implant irritation occurred in one dog. In complicated cases requiring implant removal this was performed after radiographic confirmation of osteotomy healing and typically occurred during the early postoperative follow-up period, prior to the final eight-week clinical evaluation. All dogs requiring implant removal showed radiographic osteotomy healing at the time of implant removal, and limb function improved following implant removal, allowing these cases to be classified as having good or excellent outcomes at the eight-week evaluation.

Complication rates did not differ significantly between unilateral and bilateral procedures (25.9% vs. 34.8%, respectively; *p* = 0.381; Table 1). No statistically significant associations were identified between other patient-related variables, including age and body weight, and the occurrence of complications or clinical outcome (*p* > 0.05 for all comparisons). Bone graft use was significantly associated with a lower incidence of complications (*p* = 0.008), with complications observed in 2 of 14 grafted stifles and 33 of 63 non-grafted stifles (Table 1). No complications were directly attributable to the bone graft material itself. The two complications observed in grafted stifles consisted of minor implant-related skin irritation and resolved after conservative management or implant removal. In contrast, complications in non-grafted stifles consisted primarily of implant-related skin irritation associated with the screw or pin and occasional wound complications.

Minor complications consisted of transient soft tissue irritation that resolved with conservative management. without further surgical intervention. Mean lameness score improved significantly from 3 (severe lameness) preoperatively to 1 (mild lameness) at eight weeks postoperatively, reflecting restoration of stifle stability and progressive functional recovery following tibial tuberosity advancement (paired *t*-test, *p* < 0.001). At eight weeks, 50 of 63 dogs (79%) were classified as having an excellent outcome, while the remaining 13 dogs (21%) achieved a good outcome. Dogs classified as having a good outcome showed functional limb use with only mild or intermittent lameness, without major complications affecting overall recovery. Early intervention in cases with major postoperative complications resulted in complete resolution of the problem and no evidence of functional impairment was observed at the 8 week follow-up clinical examination in these cases. In dogs requiring implant removal, radiographic healing had already been confirmed, and limb function improved after implant removal, allowing these cases to be classified as having good or excellent outcomes at the final evaluation.

No acute surgical site infections were diagnosed and all findings were based on clinical assessment without microbiological confirmation. All implant removals were performed due to implant-related skin irritation caused by prominence of the screw head or pin beneath the skin or wound complications, without clinical or radiographic evidence of infection.

The use of bone graft had a positive effect on outcome (Table 2). Overall, excellent outcomes were seen in 50/63 dogs, (79.4%), while excellent outcomes were observed in 12/14 grafted stifles (85.7%). Bone graft use was significantly associated with a more favorable outcome classification (*p* = 0.0003; Table 2).

**Figure 1:**
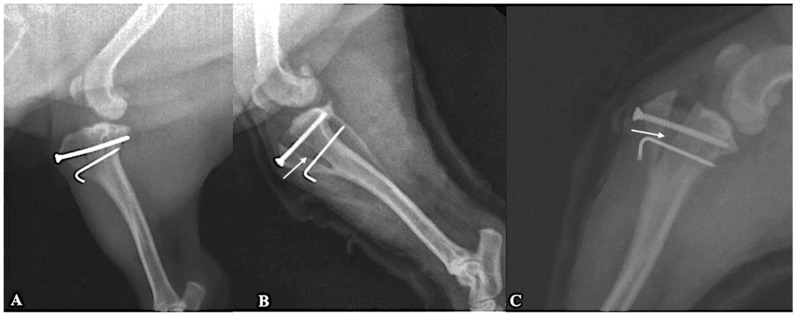
Mediolateral radiographs obtained eight weeks postoperatively showed treated stifle joints following cageless tibial tuberosity advancement. (**A**) Stifle joint stabilized with a screw–pin construct and bone graft substitute, demonstrating stable implants and advanced bone healing at the osteotomy site (healing grade 3). (**B**) Stifle joint stabilized with a screw–pin construct without bone graft substitute; white arrows indicate areas of early bone bridging across the osteotomy gap consistent with healing grade 2. (**C**) Stifle joint stabilized with a screw–pin construct and bone graft substitute, white arrows indicate areas of early bone bridging across the osteotomy gap consistent with healing grade 2.

The degree of tibial tuberosity advancement was determined preoperatively using the common tangent method; however, advancement magnitude was not quantitatively correlated with radiographic healing scores in the present retrospective study (Figure 1). Healing scores of 2 or 3 were recorded in all cases. No construct collapse, implant failure, or clinically relevant loss of advancement was identified during the follow-up period.

## 4. Discussion

The principal findings of this study were a favorable short-term clinical outcome, satisfactory radiographic healing, low rates of major complications, and an apparent beneficial association of bone graft use with both complications and outcome. Favorable short-term clinical outcomes were observed in small-breed dogs treated with a cageless tibial tuberosity advancement technique. Of the 77 operated stifles, 65 were stabilized using a screw–pin construct, 10 using screw-only fixation, and 2 using a screw combined with two pins. Due to the small size of the latter subgroups, outcome comparisons between fixation types were interpreted descriptively.

Of the 63 dogs included, 79% achieved an excellent outcome and the remaining 21% achieved a good outcome at eight weeks postoperatively. These findings are comparable to previously reported short-term outcomes following tibial tuberosity advancement procedures in small-breed dogs, particularly the TTA Rapid technique, which has also been associated with high owner satisfaction and low complication rates [6,8,9,24]. The similarity of these results suggests that the simplified screw-based fixation used in the present study may provide clinical improvement comparable to other modified TTA techniques in small dogs.

Radiographic evaluation confirmed osteotomy healing in all cases at eight weeks postoperatively, with no clinical or radiographic evidence of instability, construct collapse, or loss of tibial tuberosity advancement during the early healing period. Although immediate postoperative advancement was not quantitatively compared with follow-up measurements, no clinically relevant changes were identified. Ideally, advancement should be measured on immediate postoperative radiographs and compared with follow-up images to assess maintenance of advancement over time. Thermal bone injury adjacent to the osteotomy combined with quadriceps muscle forces may theoretically contribute to loss of advancement during healing. However, comparative studies specifically designed to assess advancement maintenance following tibial tuberosity advancement are limited, and further investigation is warranted across different TTA techniques. Similar to previous reports of modified TTA techniques, osteotomy healing was observed radiographically in all cases by the eight-week evaluation [22,25,26,27]. In addition, no clinically relevant loss of advancement was detected, which is in agreement with reports describing satisfactory short-term stability of modified TTA constructs in small dogs [9,13,17].

In small-breed dogs, the relatively short osteotomy length and limited tibial tuberosity bone stock may complicate accurate placement of conventional TTA cages and plates, particularly when implants are not specifically designed for small patients. In this case series, fixation using a single screw, with or without an additional distal pin, was sufficient to maintain advancement throughout the healing period. The potential advantages of this simplified technique include a reduced number of implants, elimination of specialized cages, and procedural simplicity. Although the magnitude and direction of forces acting on the advanced tibial tuberosity in small-breed dogs remain poorly characterized, fixation failure was not observed. Nevertheless, as load transfer is concentrated through a single screw, careful attention to accurate screw placement is essential. Whereas conventional TTA techniques commonly use cages and plates, several modified procedures have attempted to reduce implant number and surgical complexity [12,13,14,15,16,17,18,19]. The absence of fixation failure in the present study suggests that, in small-breed dogs, screw-based fixation may provide sufficient stability despite using fewer implants than many previously described systems.

The incidence of meniscal injury in this study (9.1%) was lower than previously reported rates ranging from 15% to 48% [6,8,25,26,27,28]. This finding may be influenced by the retrospective study design, potential differences in case selection or diagnostic approach, and the relatively short follow-up period, which may have limited the detection of late meniscal injury. No statistically significant association between meniscal injury and the evaluated variables (age, body weight, laterality, or bone graft use) was identified, and the cause of the lower incidence remains unclear. Meniscal release was not performed in any case, and no meniscal tears were identified during the eight-week follow-up period. However, the relatively short follow-up duration limits the ability to detect late meniscal tears. This difference may reflect variation in case selection, diagnostic approach, or the relatively short follow-up period of the present study. This conservative approach reflects surgeon preference and was supported by the low incidence of meniscal complications observed.

The Maquet hole has been proposed to reduce fissure formation; however, its efficacy remains inconsistent, and fissure propagation from the tunnel has been reported [13,19,28]. In the present study, the Maquet hole was omitted, and no fissures extending from the distal osteotomy were identified. There is currently no consensus regarding the necessity of a Maquet hole in small-breed dogs, and variability among published populations limits direct comparison [29]. In agreement with some previous reports questioning the routine necessity of a Maquet hole, no fissures extending from the distal osteotomy were identified in the present series despite omission of this step [13,19,28,30].

Placement of a bone graft substitute within the osteotomy gap was associated with a lower complication rate and more favorable clinical outcomes. This observation may be partially explained by the osteoconductive properties of calcium phosphate bioceramics, which provide a scaffold for new bone formation and may enhance early stability of the osteotomy gap, potentially reducing micromotion and implant-related irritation during the early healing phase. Therefore, the observed association between bone graft use and improved outcomes should be considered exploratory and interpreted with caution.

Complications were uncommon in grafted stifles and occurred significantly less frequently than in non-grafted stifles. Implant-related skin irritation and subsequent implant removal were the most common complications in the non-grafted group. These complications likely resulted from mechanical irritation caused by implant prominence beneath relatively thin soft tissue coverage in small-breed dogs rather than from infection. Radiographic union was confirmed in all cases requiring implant removal. Although the beneficial effects of bone grafting on bone healing are well documented [28,29,31], previous studies have reported variable effects on radiographic healing and complication rates [31,32,33]. The beneficial association observed in grafted stifles is consistent with previous studies reporting improved radiographic healing or reduced complications when graft material was used in TTA procedures [29,31,32,33], although not all published reports have demonstrated a uniform benefit. In the present study, grafted stifles had fewer complications and more favorable clinical outcomes, which may reflect enhanced early osteotomy stability and bone formation. In the present study, bone graft use was associated with improved clinical outcomes; however, the small number of grafted cases and the non-standardized use of graft material limit definitive conclusions, and this finding should be interpreted with caution.

Radiographic examples illustrate maintained advancement during early healing in both grafted and non-grafted stifles (Figure 1). Standardized radiographic positioning would be required for accurate quantitative assessment of subtle changes in advancement over time.

No surgical site infections were observed during the eight-week follow-up period. Infection status was determined based on clinical and radiographic evaluation, and bacterial culture and sensitivity testing were not routinely performed. This compares favorably with previously reported infection rates for TTA procedures using metallic implants [17,33,34,35,36,37]. While late infections cannot be excluded due to the short follow-up period, the reduced number of implants may contribute to a lower infection risk. All implant removals were performed due to implant-related skin irritation or wound complications, without clinical or radiographic evidence of infection. We classify implant removal as a major complication as implant removal has clinical relevance in that additional procedures are required which may influence patient recovery and case management.

Simultaneous bilateral orthopedic procedures may offer advantages including reduced overall convalescence time and lower costs for owners [38]. Previous studies have reported comparable outcomes and complication rates between unilateral and bilateral procedures [38,39,40,41,42,43]. In the present study, complication rates did not differ between unilateral and bilateral surgeries, although larger studies are required to further evaluate this finding. These factors may have influenced the strength and interpretation of the observed associations, and therefore the results should be interpreted as exploratory rather than confirmatory.

This study has several limitations. The retrospective design and relatively short follow-up period restrict conclusions to short-term clinical and radiographic outcomes, and late complications or long-term maintenance of advancement could not be assessed. In addition, progression of osteoarthritis was not evaluated radiographically or clinically, which limits assessment of the potential long-term effects of the procedure on joint degeneration.

The absence of a control group and objective gait analysis limits comparison with other techniques. In addition, inconsistent radiographic positioning prevented precise measurement of tibial tuberosity advancement over time. Owner-reported outcome assessment without a validated questionnaire may have introduced bias. The small number of grafted cases limits the strength of conclusions regarding bone graft use.

Additional limitations of this retrospective study are the use of different fixation methods and the variability in the use of bone grafts. Both of these factors introduce heterogeneity which may potentially influence the outcome. The use of a distal pin and bone graft were based on the surgeon’s discretion, and may have introduced confounding factors influencing the observed outcomes and complication rates. These decisions were likely influenced by patient-specific factors or intraoperative considerations that were not controlled for in the analysis. Although the majority of cases were treated using a screw–pin construct, a small number of stifles were stabilized using screw-only fixation or screw combined with two pins. These constructs likely have different biomechanical characteristics; however, their small numbers prevented meaningful statistical comparison. Sensitivity analysis excluding these cases produced results consistent with the overall analysis, suggesting that the primary findings of the study remain representative of the predominant screw–pin technique. Additionally, some dogs contributed bilateral stifles, and therefore the assumption of independence between observations may not have been fully met. This may have introduced a degree of clustering at the patient level that was not accounted for in the statistical analysis. However, this variability reflects real-world clinical conditions and may enhance the external validity of the findings. In addition, the statistical analysis was primarily univariate and did not account for potential confounding factors. Variables such as bone graft use and fixation strategy were not standardized and were determined intraoperatively, which may have influenced the observed associations with outcomes and complication rates. Therefore, the reported associations should be interpreted with caution and not as evidence of causal relationships.

Postoperative rehabilitation was not standardized, and variability in rehabilitation protocols and owner compliance may have influenced functional recovery and clinical outcomes. The absence of objective gait analysis limits direct comparison with studies that have used force plate or pressure-sensitive walkway systems to quantify limb loading and functional recovery, as well as investigations exploring morphometric and biomechanical influences on surgical outcomes.

Although all procedures were performed by experienced surgeons, which represents a strength in terms of technical consistency, this may limit the generalizability of the results to less experienced clinicians or different clinical environments.

These limitations are inherent to retrospective clinical studies and should be considered when interpreting the findings.

## 5. Conclusions

In this retrospective case series, cageless TTA using screw-based fixation (predominantly screw–pin constructs) was associated with favorable short-term clinical and radiographic outcomes in small-breed dogs. However, these findings should be interpreted with caution given the retrospective design, lack of standardization of certain intraoperative variables, and short follow-up period. Further prospective studies with larger populations, standardized outcome measures, and longer follow-up are warranted to confirm these results.

## Figures and Tables

**Table 1:** Variables associated with postoperative complications (per-stifle analysis; *n* = 77).

Variable	Category	No Complication (*n*)	Complication (*n*)	*p*-Value
Laterality	Unilateral	40	14	0.381
	Bilateral	15	8	
Bone graft	No	30	33	0.008
	Yes	12	2	

Data are presented as the number of stifles (*n*). Statistical significance was set at *p* < 0.05.

**Table 2:** Variables associated with outcome quality.

Variable	Category	Good Outcome (*n*)	Excellent Outcome (*n*)	*p*-Value
Bone graft	No	13	50	0.0003
	Yes	2	12	

Data are presented as numbers (*n*); statistical significance was set at *p* < 0.05. Complications were assessed per stifle (*n* = 77). Percentages are calculated per stifle.

## Data Availability

The original contributions presented in this study are included in the article/Supplementary Material. Further inquiries can be directed to the corresponding author.

## Supplementary Materials

The following supporting information can be downloaded at: https://www.mdpi.com/article/10.3390/ani16081212/s1, Table S1: Detailed individual case data for the 63 dogs (77 stifles) included in the study.
