# Correction: McCartney et al. Retrospective Clinical and Radiographic Outcomes of a Cageless Tibial Tuberosity Advancement Technique in Small-Breed Dogs. *Animals* 2026, *16*, 1212

**DOI:** 10.3390/ani16111701

**Published:** 2026-06-02

**Authors:** William McCartney, Christos Yiapanis, Ciprian Ober, Amarildo Gjeli, Denis Gaceu, Joshua Milgram

**Affiliations:** 1 North Dublin Orthopaedic Animal Hospital, Warrenhouse Rd 38, Baldoyle, D13 K5H0 Dublin, Ireland; billymccartney@gmail.com; 2 Department of Veterinary Medicine, School of Veterinary Medicine, University of Nicosia, 2414 Nicosia, Cyprus; drcy@cyvets.com; 3 Department of Surgery and Intensive Care, Faculty of Veterinary Medicine, University of Agricultural Sciences and Veterinary Medicine, Calea Manastur 3-5, 400372 Cluj-Napoca, Romania; gaceudenis@gmail.com; 4 CYVETS, Leof. T. Papadopoulou 138, 8025 Paphos, Cyprus; amarildogj@cyvets.com; 5 Department of Small Animal Surgery, Koret School of Veterinary Medicine, The Robert H. Smith Faculty of Agriculture, Food & Environment, The Hebrew University of Jerusalem, P.O. Box 12, Rehovot 76100, Israel; josh.milgram@mail.huji.ac.il

## Reference Correction

There was an error in Reference 21 in the original publication [1]. The incorrect reference has been replaced with the following correct reference. 

The incorrect reference:

Della Valle, G.; Caterino, C.; Aragosa, F.; Micieli, F.; Costanza, D.; Di Palma, C.; Fatone, G. Objective gait analysis in dogs undergoing surgical treatment for cranial cruciate ligament disease: A review of methods and outcomes. *Vet. Comp. Orthop. Traumatol.* **2021**, *34*, 1–10.

has been replaced with the following correct reference:

Della Valle, G.; Caterino, C.; Aragosa, F.; Micieli, F.; Costanza, D.; Di Palma, C.; Piscitelli, A.; Fatone, G. Outcome after Modified Maquet Procedure in dogs with unilateral cranial cruciate ligament rupture: Evaluation of recovery limb function by use of force plate gait analysis. *PLoS ONE* **2021**, *16*, e0256011. https://doi.org/10.1371/journal.pone.0256011.

The authors state that the scientific conclusions are unaffected. This correction was approved by the Academic Editor. The original publication has also been updated.
