# Supplementary material for: Retrospective Clinical and Radiographic Outcomes of a Cageless Tibial Tuberosity Advancement Technique in Small-Breed Dogs
Source: Animals (Basel). 2026 Apr 16;16(8):1212. doi: 10.3390/ani16081212 (PMC13113057; doi:10.3390/ani16081212)
Supplement: Supplementary file 1 [file animals-16-01212-s001.zip › animals-4217902-supplementary.pdf]

Table S1. Signalment and implant configuration.

No.	Breed	Age (years)	Sex	Weight (kg)	Implants: screw 2,4 mm/pins 1.1-1.6mm	Bone graft	Complications	Comment	Outcome
1	Terrier Cross	8	Fn	7	screw and pin	no	none		excellent
2	Whippet	4	Fn	6	screw and pin	no	wound breakdown implants removed		good
3	French bull dog	4	F	14	screw and pin	yes	none		excellent
4	Poodle	8	Fn	9	screw	no	none		excellent
5	Poodle	15	Mn	9	screw and pin	no	screw removal due to irritation screw and pin		excellent
6	Jack Russell	8	Mn	9	screw and pin	no	none		excellent
7	Cross breed	11	Fn	10	screw and pin	no	none		excellent
8	Frise	10	Fn	10	screw and pin	no	pin irritation	Bilateral same time	excellent
9	Terrier X	5	Fn	12	screw and pin	no	pin irritation (pin removed)		good
10	Bichon Frise	4	Fn	7	screw and pin	yes	none	Bilateral same time	good
11	Shih tzu	11	Fn	11	screw and pin	yes	none		good
12	Shih tzu	5	Fn	7	screw and pin	yes	none	Bilateral same time	excellent
13	Terrier Cross	14	F	9	screw and pin	no	none		excellent
14	Shih tzu	7	Mn	9	screw and pin	no	screw irritation (not removed)		excellent
15	Terrier Cross	9	Fn	3	screw and pin	yes	none		excellent
16	Terrier Cross	8	Fn	7	screw and pin	no	none		excellent
17	Pinscher Cross	8	Fn	6	screw	no	screw removal due to irritation		excellent
18	Cocker spaniel	5	Mn	13	screw and pin	yes	none	Bilateral staged	excellent
19	Chihuahua	10	Mn	4.7	Left: screw, Right: screw and pin	no	none	Bilateral staged	excellent

https://doi.org/10.3390/xxxxx

20	Terrier X	6	Fn	4	screw and pin	no	none		excellent
21	Terrier X	4	Fn	7	screw and pin	yes	none		good
22	Bichon Frise	5	M	9	screw and pin	no	screw irritation (not removed)		excellent
23	Terrier x	10	M	11	screw and pin	no	none		excellent
24	Cavachon	5	Mn	11	screw and pin	yes	none		excellent
25	French bulldog	3	Mn	11	screw and pin	no	screw irritation (not removed)	Bilateral same time	excellent
26	Cavachon	8	Fn	5	screw and pin	no	none		excellent
27	Hound	8	Fn	9.5	screw and two pin	no	none		good (working dog)
28	Maltese	8	M	6	screw	no	none		excellent
29	West Highland white terrier	9	Fn	7	screw and pin	no	pin irritation (pin removed)		excellent
30	Bichon Frise	9	Mn	5	screw and pin	yes	none	Bilateral same time	excellent
31	Bichon Frise	9	Mn	4	screw and pin	no	none		excellent
32	Shih tzu	9	M	7	screw and pin	no	none		excellent
33	Lhasa Apso	9	Mn	9	screw and pin	no	none	Bilateral same time	excellent
34	Beagle	9	Fn	10	Left screw and pin, Right: screw.	no	none	Bilateral staged	excellent
35	Bichon frise	13	Fn	7	screw and pin	yes	none		excellent
36	Terrier X	11	Fn	5	screw and pin	yes	none		excellent
37	Beagle	8	Fn	10	screw	no	screw removal due to irritation		excellent
38	Terrier Cross	9	Mn	10	screw and pin	no	screw removal due to irritation screw and 2 pins	Bilateral staged	excellent
39	Shih tzu	12	Fn	11	screw and pin	yes	none		good
40	Hound	7	Fn	9	screw and two pins	no	none		good (working dog)
41	Chihuahua	11	Fn	6	screw and pin	no	none		excellent
42	Jack Russell	12	Fn	13	screw and pin	yes&no	none	Bilateral staged	excellent

https://doi.org/10.3390/xxxxx

43	Cross	11	F	9	screw and pin for both. 2nd leg	no	none	Bilateral staged	excellent
44	Terrier x	8	Mn	10	screw and pin	no	none	Bilateral staged	excellent
45	Patterdale	6	M	8	screw and pin	no	none		excellent
46	Terrier	6	Fn	8	screw and pin	no	none		excellent
47	Minature snau-zer	9	Fn	8	screw and pin	no	none	Bilateral staged	excellent
48	Bichon frise	5	Fn	8	screw and pin	yes	none		good
49	Tibetan Spaniel	12	Mn	9	screw and pin	no	pin irritation (pin removed)		excellent
50	Cavachon	6	Fn	12	screw and pin	no	screw irritation (not removed)		excellent
51	Minature Snau-zer	4	Fn	8	screw and pin	no	none		excellent
52	Pomeranian	6	Fn	5	screw	no	none		excellent
53	Lhasa Apso	12	Fn	8	screw and pin	no	none		excellent
54	West Highland white terrier	9	Fn	9	screw and pin	yes	none	Bilateral same time	excellent
55	Jack Russell	12	Mn	10	screw and pin	no	none		excellent
56	Poodle	11	M	9	screw	no	screw removal due to irritation		excellent
57	Terrier	4	F	6	screw and pin	no	none		excellent
58	Jack Russell	13	Mn	13	screw and pin	no	none		good
59	Hungarian wire hair	3	M	11	screw and pin	no	none		excellent
60	Cockapoo	3	M	8	screw and pin	no	none		excellent
61	Bichon frise	6	Fn	5	screw and pin	no	none		excellent
62	Poodle	12	Fn	10	screw only for both	no	none	Bilateral staged	excellent
63	Shih Tzu	7	M	6	screw and pin	no	none		excellent

Data are presented per dog unless otherwise specified. In total, 63 dogs contributed 77 stifles; 49 dogs underwent unilateral surgery and 14 dogs underwent bilateral surgery (8 simultaneous and 6 staged). Abbreviations: Fn, female neutered; Mn, male neutered
